# Supplementary material for: Cholesteryl ester transfer protein (CETP) as a drug target for cardiovascular disease
Source: Nat Commun. 2021 Sep 24;12:5640. doi: 10.1038/s41467-021-25703-3 (PMC8463530; doi:10.1038/s41467-021-25703-3)
Supplement: Supplementary file 1 — Supplementary Information [file 41467_2021_25703_MOESM1_ESM.pdf]

Supplementary Information: Cholesteryl Ester Transfer Protein (CETP) as a Drug Target

A F Schmidt et al

Contents

Supplementary Figures 1

Supplementary Tables 6

Supplementary note 1 23

Supplementary note 2 26

Supplementary references 27

Supplementary Figures

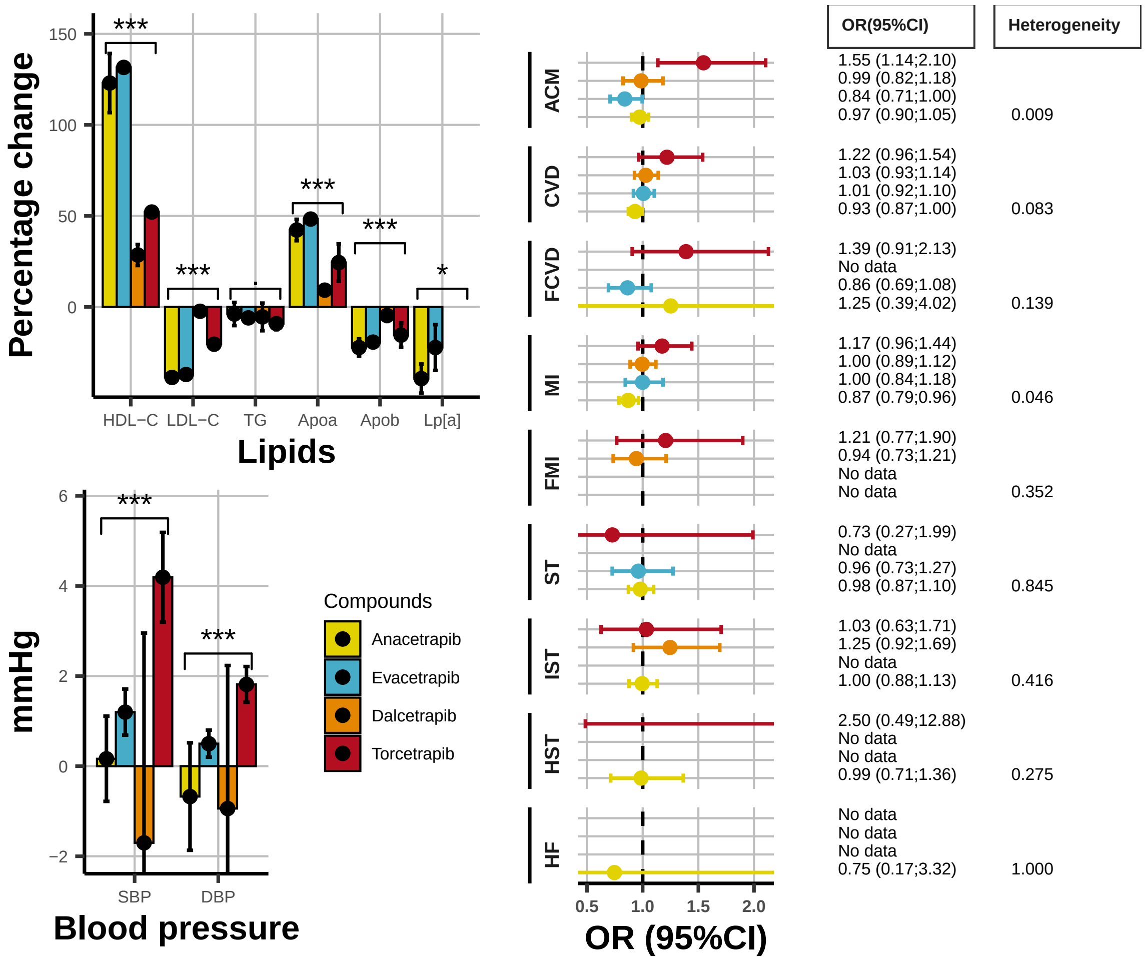

Supplementary Figure 1: Random effects meta-analyses of CETP inhibitors. The difference column presents a p-value for the difference in OR across the 4 compounds. Similarly; *** heterogeneity p-value < 0.001, ** 0.01 < p-value < 0.001, * 0.05 < p-value < 0.01, . p-value < 0.05. Abbreviations: LDL-C, low-density lipoprotein cholesterol; HDL-C, high-density lipoprotein cholesterol; TG, triglycerides; ApoB, apolipoprotein B; ApoA1, apolipoprotein A1; Lp[a], lipoprotein[a]; SBP, systolic blood pressure; DBP, diastolic blood pressure; ACM, all-cause mortality; CVD, any cardiovascular disease; FCVD, fata-CVD; MI, any myocardial infarction; FMI, fatal-MI; ST, stroke; IST, ischemic ST; HST, hemorrhagic ST; HF, heart failure. The error bars represent 95% confidence intervals (95%CI), where the central dot depicts the odds ratio (LHS) or mean difference (RHS). The number of subjects and events are listed in table 3.

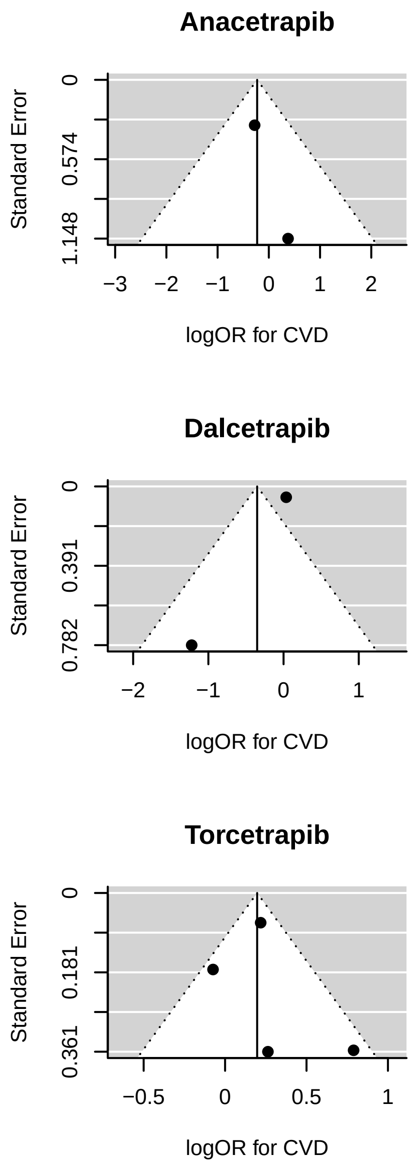

Supplementary Figure 2: Funnel plots assessing small sample treatment heterogeneity in cardiovascular (CVD) outcomes of CETP-inhibitors with more than one included trial (excluding the single study on Evacetrapib).

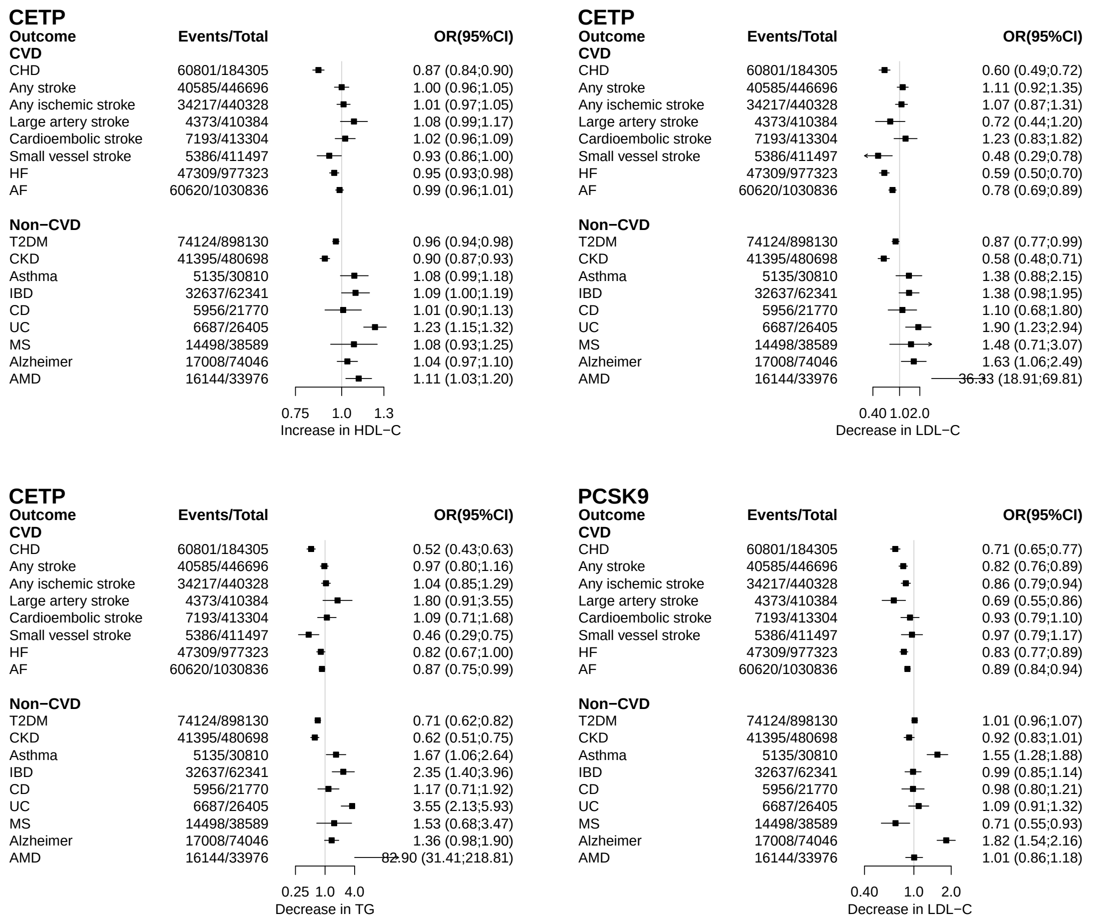

Supplementary Figure 3: Drug target MR effects of CETP and PCSK9 clinically relevant outcomes proxied by the genetic effects on LDL-C (for *CETP* and *PCSK9*), HDL-C and TG (for *CETP*). Results are coded in the canonical, treatment direction (HDL-C *increase*, LDL-C and TG *decrease*). The error bars represent 95% confidence intervals (95%CI), where the central dot depicts the odds ratio. The number of events and total samples are depicted in the figure as ”Events/Total”.

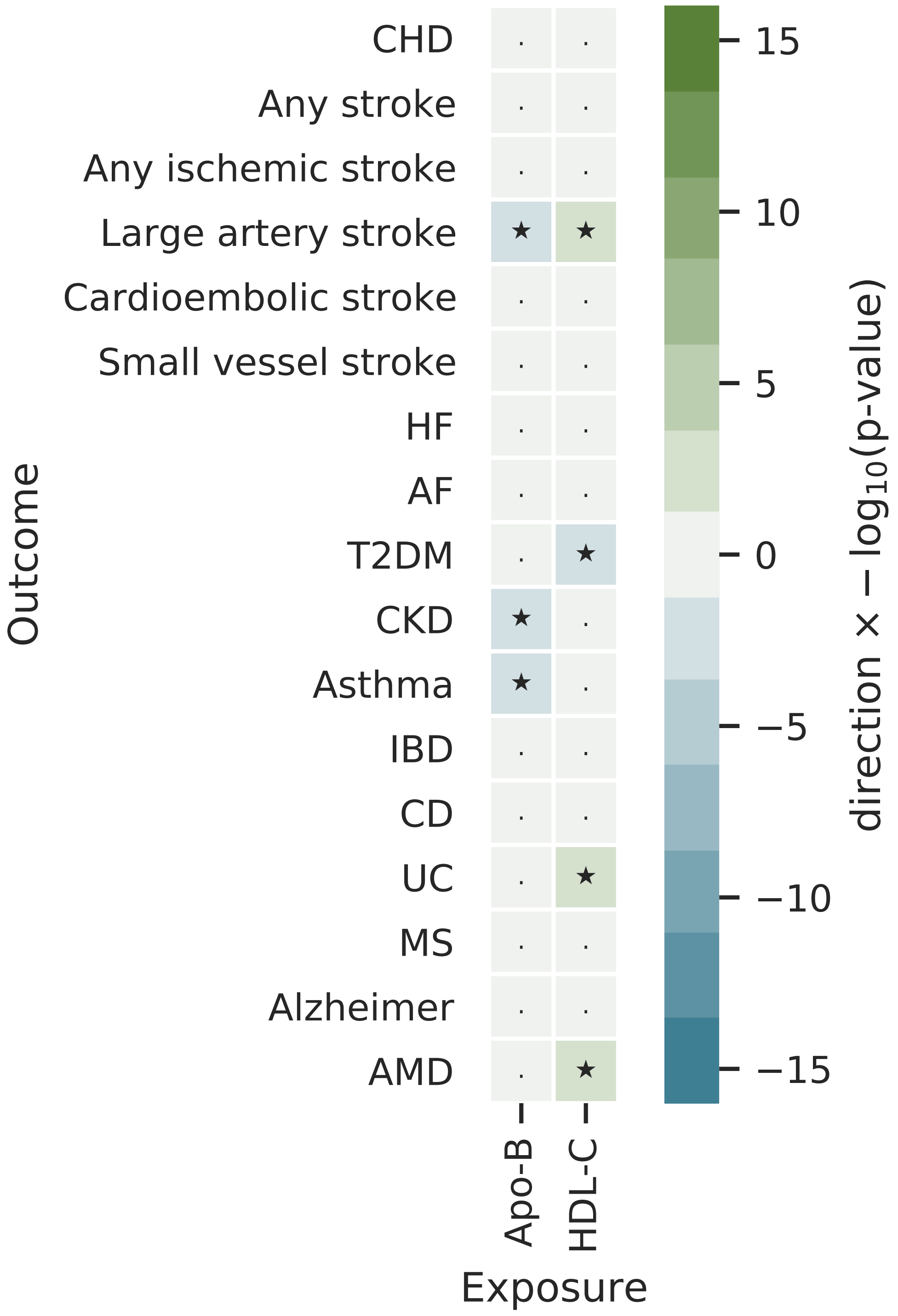

Supplementary Figure 4: Multivariable drug target MR assessing the potential Apo-B and HDL-C mediation pathway of the CETP effects on clinical outcomes. Colours indicate effect direction multiplied by $-\log_{10}$ p-value, which was truncated at ± 16. Results are coded in the canonical, treatment direction (HDL-C *increase*, Apo-B *decrease*). Stars indicate p-value less than 0.05.

Supplementary Tables

Supplementary Table 1: CETP compounds evaluated in clinical trials

Compound	Maximum clinical development phase	Continued development
Anacetrapib	3	Terminated
ATH03	1	Terminated
Dalcetrapib	3	Ongoing
DRL-17822	2	Terminated
Evacetrapib	3	Terminated
JTT-302	2	Terminated
K-312	1	Terminated
Obicetrapib	2	Ongoing
Rocacetrapib	2	Terminated
Torcetrapib	3	Terminated

Supplementary Table 2: CETP inhibitor trials

Trial	Pubmed ID	Participants	CETP compound	Comparison	Background therapy	CETP dose (mg)	Median follow-up (days)	No. of subject in the inter-vention group	No. of subject in the com-parator group
ARAI	27131642	FH patients	Anacetrapib	Placebo	Any Kind Of Statin + Potential Other Lipid Lowering Therapies	100	168	34	34
REVEAL	28847206	Patients with atherosclerotic vascular disease	Anacetrapib	Placebo	Atorvostatin	100	1497	15225	15224
REALIZE	25743173	FH patients	Anacetrapib	Placebo	Any Kind Of Statin Or Ezetimbe	100	448	204	102
DEFINE	21082868	Patients with coronary heart disease or at high risk for coronary heart disease	Anacetrapib	Placebo	Any Kind Of Statin Or Ezetimbe	100	532	811	812
TERAMOTO	28478132	High risk dyslipidemia patients	Anacetrapib	Placebo	Any Kind Of Statin + Potential Other Lipid Lowering Therapies	100	364	204	103
BALLENTYNE	28624096	Hypercholesterolemia subjects, not meeting treatment goals.	Anacetrapib	Placebo	Any Kind Of Statin + Potential Other Lipid Lowering Therapies	100	252	290	293
DAL-PLAQUE	21908036	Subjects with a history of CHD or a high risk of CHD	Dalcetrapib	Placebo	Any Kind Of Statin + Potential Other Lipid Lowering Therapies	600	168	64	66
STEIN	20097702	Subjects with a history of CHD or a high risk of CHD	Dalcetrapib	Placebo	Atorvastatin	900	336	89	56
DAL-OUTCOMES	23126252	Patients with a recent acute coronary syndrome	Dalcetrapib	Placebo	Best Available Care	600	943	7938	7933
DAL-VESSEL	22345126	Patients with CHD or CHD risk equivalent and HDL-C below 50 mg/dL	Dalcetrapib	Placebo	Any Kind Of Statin Or Ezetimbe	600	252	238	238
RADIANCE 1	17387131	FH patients	Torcetrapib	No CETP-I	Atorvastatin	60	730	450	454
RADIANCE 2	17630038	Subjects with mixed dyslipidaemia	Torcetrapib	No CETP-I	Atorvastatin	60	548	377	375
ILLUSTRATE	17387129	Patients with coronary disease	Torcetrapib	No CETP-I	Atorvastatin	60	730	591	597
ILLUMINATE	17984165	Patients with a history of cardiovascular diseas	Torcetrapib	No CETP-I	Atorvastatin	120	550	7533	7534
ACCELERATE	28514624	High risk patients with a history of ACS, PAD, or T2DM combined with coronary disease	Evacetrapib	Placebo	Any Kind Of Statin Or Other Lipid Lowering Therapies	130	791	6038	6054

Note:

FH, familial hypercholesterolaemia

Supplementary Table 3: Compound specific effects of CETP inhibition on lipids, lipoporteins, blood pressure, and clinical endpoints

	Anacetrapib				Dalcetrapib				Torcetrapib				Evacetrapib				
	No. Trials	No. events	No. subjects	OR/MD 95%CI	No. Trials	No. events	No. subjects	OR/MD 95%CI	No. Trials	No. events	No. subjects	OR/MD 95%CI	No. Trials	No. events	No. subjects	OR/MD 95%CI	Heterogeneity p-value
LDL-C (% change)	6	0	30636	-37.89 (-39.76;-36.03)	4	0	16366	-1.00 (-1.06;-0.94)	4	0	17510	-20.46 (-23.54;-17.37)	1	0	12092	-37.10 (-38.11;-36.09)	< 0.01
HDL-C (% change)	6	0	30647	129.86 (126.88;132.85)	4	0	16366	28.55 (22.77;34.32)	4	0	17510	52.15 (49.18;55.12)	1	0	12092	131.60 (130.09;133.11)	< 0.01
Triglycerides (% change)	6	0	30647	-2.98 (-5.12;-0.83)	4	0	13273	0.03 (-0.93;0.98)	4	0	17467	-8.56 (-11.12;-6.01)	1	0	12092	-6.00 (-6.37;-5.63)	< 0.01
Apolipoprotein A1 (% change)	6	0	30672	42.70 (41.24;44.16)	3	0	16363	9.25 (6.19;12.31)	2	0	15917	24.39 (14.11;34.66)	1	0	12092	48.30 (47.35;49.24)	< 0.01
Apolipoprotein B (% change)	6	0	30673	-20.40 (-21.94;-18.85)	1	0	415	-4.68 (-7.98;-1.38)	2	0	15917	-15.47 (-22.17;-8.77)	1	0	12092	-19.30 (-20.09;-18.51)	< 0.01
Lipoportein a (% change)	6	0	30663	-40.76 (-43.96;-37.56)	0	0	0	NA (NA;NA)	0	0	0	NA (NA;NA)	1	0	12092	-22.30 (-34.86;-9.74)	< 0.01
Systolic blood pressure (mm Hg)	4	0	32891	0.57 (0.14;1.01)	2	0	553	-1.27 (-2.29;-0.24)	4	0	17567	4.44 (4.33;4.54)	1	0	12092	1.20 (0.69;1.71)	< 0.01
Diastolic blood pressure (mm Hg)	4	0	32897	0.17 (-0.09;0.43)	2	0	553	-0.29 (-0.98;0.40)	4	0	17567	2.03 (1.95;2.12)	1	0	12092	0.50 (0.20;0.80)	< 0.01
All-cause mortality	3	2300	33014	0.97 (0.90;1.05)	1	455	16478	0.99 (0.82;1.18)	3	168	17007	1.55 (1.14;2.10)	1	507	12092	0.84 (0.71;1.00)	< 0.01
Any CVD	3	3149	33014	0.93 (0.87;1.00)	3	1312	16343	1.03 (0.93;1.14)	4	1021	17911	1.22 (1.08;1.38)	1	1555	12092	1.01 (0.92;1.10)	< 0.01
Fatal CVD	2	1089	32711	0.93 (0.82;1.04)	0	0	0	NA (NA;NA)	2	87	17159	1.39 (0.91;2.13)	1	309	12092	0.86 (0.69;1.08)	0.15
Fatal and non-fatal MI	1	1454	32711	0.87 (0.79;0.96)	2	1070	16343	1.00 (0.89;1.12)	2	363	16255	1.17 (0.96;1.44)	1	517	12092	1.00 (0.84;1.18)	0.05
Fatal MI	0	0	0	NA (NA;NA)	1	244	16343	0.94 (0.73;1.21)	2	75	16255	1.21 (0.77;1.90)	0	0	0	NA (NA;NA)	0.35
Any stroke	2	1118	32711	0.98 (0.87;1.10)	0	0	0	NA (NA;NA)	3	95	17159	0.97 (0.64;1.46)	1	192	12092	0.96 (0.73;1.27)	0.99
Ischemic stroke	1	922	30517	1.00 (0.88;1.13)	1	164	16343	1.25 (0.92;1.69)	1	61	15067	1.03 (0.63;1.71)	0	0	0	NA (NA;NA)	0.42
Hemorrhagic stroke	1	145	30517	0.99 (0.71;1.36)	0	0	0	NA (NA;NA)	1	7	15067	2.50 (0.49;12.88)	0	0	0	NA (NA;NA)	0.28
Heart Failure	1	7	1612	0.75 (0.17;3.32)	0	0	0	NA (NA;NA)	0	0	0	NA (NA;NA)	0	0	0	NA (NA;NA)	1.00

Note:

Estimates represent fixed effects. OR, odds ratio; MD, mean difference; 95%CI, 95% confidence interval

Supplementary Table 4: Meta-regression of CETP-inhbitor trial-specific baseline characteristics or compound effects on lipid biomarkers.

	Anacetrapib		Dalcetrapib		Torcetrapib	
	No. Trials	OR 95%CI	No. Trials	OR 95%CI	No. Trials	OR 95%CI
Proportion of women	2	0.71 (0.21;2.39)	2	0.69 (0.44;1.09)	4	1.01 (0.99;1.03)
Mean Age	2	0.88 (0.55;1.40)	2	0.32 (0.08;1.29)	4	0.82 (0.67;1.00)
Mean BMI	2	Failed	2	Failed	4	2.36 (0.27;20.31)
LDL-C effect	2	Failed	2	Failed	4	0.29 (0.01;12.27)
HDL-C effect	2	Failed	2	Failed	4	0.95 (0.06;15.57)
Apo-B effect	2	Failed	1	Failed	2	Failed

Note:

Effect estimates represent the change in treatment effect as odds ratio (OR)

Supplementary Table 5: Drug target Mendelian randomization effects intermediate traits. CETP and PCSK9 effects were instrumented through genetic association with protein concentration or relevant lipid fractions

	CETP pQTL			CETP LDL-C			CETP HDL-C			CETP TG			PCSK9 pQTL			PCSK9 LDL-C		
	No. variants	MD 95%CI	heterogeneity p-value	No. variants	MD 95%CI	heterogeneity p-value	No. variants	MD 95%CI	heterogeneity p-value	No. variants	MD 95%CI	heterogeneity p-value	No. variants	MD 95%CI	heterogeneity p-value	No. variants	MD 95%CI	heterogeneity p-value
LDL-C (mmol/l)	15	-0.08 (-0.08;-0.07)	< 0.01	0	NA (NA;NA)	NA	13	-0.12 (-0.13;-0.12)	< 0.01	3	-0.66 (-0.74;-0.58)	0.27	6	-0.57 (-0.60;-0.54)	< 0.01	0	NA (NA;NA)	NA
HDL-C (mmol/l)	6	0.23 (0.23;0.24)	< 0.01	2	1.83 (1.78;1.87)	< 0.01	0	NA (NA;NA)	NA	0	NA (NA;NA)	NA	10	-0.03 (-0.04;-0.02)	0.09	11	-0.02 (-0.03;-0.01)	0.19
TG (mmol/l)	14	-0.09 (-0.10;-0.08)	0.77	7	-0.72 (-0.76;-0.67)	0.01	13	-0.15 (-0.16;-0.14)	0.01	0	NA (NA;NA)	NA	10	0.07 (0.04;0.09)	0.01	11	0.05 (0.03;0.07)	0.03
LP[a] (nmol/l)	15	-2.20 (-2.70;-1.71)	0.04	7	-9.55 (-12.15;-6.95)	0.69	13	-2.74 (-3.17;-2.32)	0.51	5	-14.05 (-16.83;-11.28)	0.97	10	-3.54 (-5.06;-2.03)	0.35	11	-2.95 (-3.98;-1.92)	0.52
ApoB (g/l)	12	-0.03 (-0.03;-0.03)	0.15	6	-0.24 (-0.25;-0.23)	0.01	11	-0.05 (-0.05;-0.04)	< 0.01	3	-0.27 (-0.28;-0.25)	0.01	6	-0.15 (-0.16;-0.14)	< 0.01	6	-0.14 (-0.14;-0.13)	0.41
ApoA1 (g/l)	8	0.13 (0.13;0.13)	< 0.01	2	1.02 (0.98;1.05)	< 0.01	5	0.19 (0.19;0.20)	< 0.01	0	NA (NA;NA)	NA	9	0.00 (-0.01;0.01)	0.13	11	0.01 (0.01;0.02)	0.01
cIMT (mm)	15	-0.00 (-0.01;-0.00)	0.77	7	-0.03 (-0.05;-0.02)	0.46	12	-0.01 (-0.01;-0.00)	< 0.01	5	-0.04 (-0.06;-0.02)	0.13	9	-0.02 (-0.03;-0.01)	0.30	11	-0.02 (-0.03;-0.02)	0.41
SBP (mmHg)	15	-0.21 (-0.33;-0.10)	0.36	7	-1.95 (-2.58;-1.31)	0.35	11	-0.39 (-0.51;-0.27)	0.03	5	-2.40 (-3.25;-1.54)	0.11	7	-1.20 (-1.70;-0.70)	0.04	10	-1.01 (-1.30;-0.72)	< 0.01
DBP (mmHg)	15	-0.12 (-0.19;-0.06)	0.74	7	-0.84 (-1.18;-0.49)	0.91	12	-0.20 (-0.26;-0.15)	0.42	5	-1.13 (-1.49;-0.77)	0.59	7	-0.32 (-0.70;0.07)	0.09	10	0.05 (-0.10;0.20)	0.01
Pulse rate (bpm)	15	-0.14 (-0.33;0.05)	0.27	7	1.18 (0.09;2.27)	0.20	12	-0.14 (-0.29;0.01)	< 0.01	5	0.24 (-0.86;1.34)	0.28	9	1.22 (0.71;1.72)	< 0.01	11	1.41 (1.05;1.77)	0.01
Glucose (mmol/l)	14	-0.02 (-0.04;-0.01)	0.23	7	-0.22 (-0.28;-0.16)	0.01	11	-0.06 (-0.08;-0.05)	0.05	5	-0.25 (-0.32;-0.19)	0.45	10	-0.03 (-0.06;0.00)	0.38	11	-0.00 (-0.03;0.03)	0.13
HbA1c (mmol/mol)	15	-0.09 (-0.15;-0.04)	0.01	7	-0.69 (-0.99;-0.39)	0.04	10	-0.24 (-0.31;-0.17)	0.01	5	-0.89 (-1.21;-0.57)	0.05	9	-0.25 (-0.43;-0.08)	0.04	11	-0.17 (-0.30;-0.04)	0.28
Leukocytes (10^9 cells/l)	15	-0.03 (-0.05;-0.01)	0.13	7	-0.31 (-0.44;-0.18)	0.07	11	-0.07 (-0.09;-0.04)	0.06	5	-0.36 (-0.47;-0.26)	0.32	10	0.09 (0.03;0.16)	0.11	11	0.07 (0.03;0.11)	0.24
Lymphocytes (10^9 cells/l)	15	-0.02 (-0.04;-0.01)	0.15	7	-0.10 (-0.15;-0.05)	0.81	13	-0.04 (-0.05;-0.03)	0.11	5	-0.16 (-0.22;-0.10)	0.77	10	0.03 (0.00;0.06)	0.02	11	0.02 (-0.00;0.04)	0.04
Monocytes (10^9 cells/l)	15	-0.01 (-0.01;-0.00)	0.01	7	-0.04 (-0.06;-0.03)	0.06	12	-0.01 (-0.01;-0.01)	0.03	5	-0.04 (-0.05;-0.03)	0.53	10	0.01 (0.00;0.01)	0.62	11	0.00 (-0.00;0.01)	0.50
Neutrophils (10^9 cells/l)	15	-0.00 (-0.02;0.01)	0.07	6	-0.10 (-0.20;-0.01)	0.20	11	-0.02 (-0.04;-0.00)	0.06	5	-0.16 (-0.26;-0.06)	0.09	10	0.05 (0.01;0.08)	0.01	11	0.04 (0.02;0.07)	0.38
CRP (mg/L)	15	0.02 (-0.02;0.06)	0.92	7	0.18 (-0.02;0.39)	0.53	13	0.03 (-0.01;0.06)	0.66	5	0.09 (-0.13;0.31)	0.89	10	0.41 (0.29;0.52)	0.01	10	0.40 (0.30;0.50)	0.01
BUN (mg/dl)	15	-0.01 (-0.01;-0.01)	< 0.01	7	-0.04 (-0.06;-0.02)	0.31	12	-0.01 (-0.01;-0.01)	0.45	5	-0.05 (-0.07;-0.03)	0.93	9	-0.04 (-0.05;-0.03)	0.01	11	-0.02 (-0.03;-0.01)	< 0.01
eGFR (SD of log(eGFR))	15	0.00 (-0.00;0.00)	0.36	7	0.01 (0.00;0.02)	0.49	12	0.00 (0.00;0.00)	0.09	5	0.01 (0.00;0.02)	0.98	8	0.02 (0.01;0.03)	0.16	10	0.01 (0.01;0.02)	0.02
BMD (SD)	15	0.01 (0.00;0.02)	0.38	7	0.10 (0.06;0.13)	0.55	11	0.01 (0.01;0.02)	0.04	5	0.10 (0.07;0.14)	0.41	6	-0.02 (-0.04;0.01)	0.01	6	0.01 (-0.01;0.03)	0.67
Cognitive function (SD)	15	-0.00 (-0.01;0.01)	0.04	6	-0.02 (-0.13;0.09)	0.71	13	-0.01 (-0.02;-0.01)	0.69	4	-0.06 (-0.12;-0.00)	0.52	9	-0.01 (-0.04;0.02)	0.70	11	-0.01 (-0.03;0.01)	0.50

Note:

NA's occurred due to a lack of coverage or, when all variants were removed due to a high risk of horizontal pleiotropy; Effects presented as mean difference (MD) and 95% confidence intervals (95%CI) per unit change in protein or lipid concentration coded in the canonical direction.

Supplementary Table 6: Drug target Mendelian randomization effects on clinical outcomes. CETP and PCSK9 effects were instrumented through genetic association with protein concentration or relevant lipid fractions

	CETP pQTL			CETP LDL-C			CETP HDL-C			CETP TG			PCSK9 pQTL			PCSK9 LDL-C		
	No. variants	OR 95%CI	heterogeneity p-value	No. variants	OR 95%CI	heterogeneity p-value	No. variants	OR 95%CI	heterogeneity p-value	No. variants	OR 95%CI	heterogeneity p-value	No. variants	OR 95%CI	heterogeneity p-value	No. variants	OR 95%CI	heterogeneity p-value
CHD	15	0.95 (0.91;0.99)	0.33	7	0.60 (0.49;0.72)	0.40	13	0.87 (0.84;0.90)	0.17	5	0.52 (0.43;0.63)	0.39	9	0.69 (0.59;0.81)	0.09	11	0.71 (0.65;0.77)	0.38
Any stroke	15	0.99 (0.95;1.02)	0.38	7	1.11 (0.92;1.35)	0.50	12	1.00 (0.96;1.05)	0.08	5	0.97 (0.80;1.16)	0.61	9	0.79 (0.69;0.91)	0.23	11	0.82 (0.76;0.89)	0.83
Any ischemic stroke	15	1.00 (0.97;1.04)	0.50	7	1.07 (0.87;1.31)	0.76	12	1.01 (0.97;1.05)	0.37	5	1.04 (0.85;1.29)	0.76	9	0.86 (0.76;0.97)	0.43	11	0.86 (0.79;0.94)	0.82
Large artery stroke	15	0.96 (0.88;1.06)	0.01	7	0.72 (0.44;1.20)	0.43	13	1.08 (0.99;1.17)	0.35	5	1.80 (0.91;3.55)	0.13	9	0.64 (0.47;0.87)	0.45	11	0.69 (0.55;0.86)	0.59
Cardioembolic stroke	15	0.95 (0.88;1.03)	0.35	7	1.23 (0.83;1.82)	0.51	13	1.02 (0.96;1.09)	0.01	5	1.09 (0.71;1.68)	0.32	9	0.76 (0.55;1.05)	0.07	11	0.93 (0.79;1.10)	0.54
Small vessel stroke	14	0.95 (0.85;1.06)	0.19	7	0.48 (0.29;0.78)	0.38	13	0.93 (0.86;1.00)	0.98	5	0.46 (0.29;0.75)	0.81	9	0.81 (0.57;1.15)	0.14	11	0.97 (0.79;1.17)	0.89
HF	15	0.96 (0.93;0.98)	0.40	7	0.59 (0.50;0.70)	0.55	13	0.95 (0.93;0.98)	0.22	5	0.82 (0.67;1.00)	0.21	9	0.79 (0.71;0.87)	0.35	11	0.83 (0.77;0.89)	0.20
AF	15	0.99 (0.96;1.01)	0.65	7	0.78 (0.69;0.89)	0.99	13	0.99 (0.96;1.01)	0.21	5	0.87 (0.75;0.99)	0.72	10	0.90 (0.83;0.97)	0.88	11	0.89 (0.84;0.94)	0.64
T2DM	15	1.00 (0.98;1.03)	0.39	7	0.87 (0.77;0.99)	0.50	13	0.96 (0.94;0.98)	0.91	5	0.71 (0.62;0.82)	0.36	9	1.10 (0.99;1.21)	0.06	10	1.01 (0.96;1.07)	0.58
CKD	15	0.94 (0.91;0.97)	0.75	7	0.58 (0.48;0.71)	0.70	13	0.90 (0.87;0.93)	0.51	5	0.62 (0.51;0.75)	0.98	9	0.83 (0.72;0.94)	0.16	11	0.92 (0.83;1.01)	0.13
Asthma	15	1.00 (0.92;1.09)	0.43	7	1.38 (0.88;2.15)	0.38	12	1.08 (0.99;1.18)	0.72	5	1.67 (1.06;2.64)	0.76	11	1.97 (1.56;2.48)	0.96	11	1.55 (1.28;1.88)	0.24
IBD	15	0.99 (0.92;1.05)	0.70	7	1.38 (0.98;1.95)	0.49	12	1.09 (1.00;1.19)	0.08	5	2.35 (1.40;3.96)	0.08	9	1.04 (0.84;1.29)	0.53	11	0.99 (0.85;1.14)	0.74
CD	15	0.94 (0.86;1.03)	0.55	7	1.10 (0.68;1.80)	0.37	12	1.01 (0.90;1.13)	0.12	5	1.17 (0.71;1.92)	0.39	9	1.25 (0.89;1.76)	0.22	11	0.98 (0.80;1.21)	0.93
UC	15	1.04 (0.95;1.13)	0.65	7	1.90 (1.23;2.94)	0.79	13	1.23 (1.15;1.32)	0.04	5	3.55 (2.13;5.93)	0.28	9	1.04 (0.80;1.36)	0.65	11	1.09 (0.91;1.32)	0.47
MS	3	1.05 (0.95;1.16)	0.91	2	1.48 (0.71;3.07)	0.90	3	1.08 (0.93;1.25)	0.95	2	1.53 (0.68;3.47)	0.97	5	0.69 (0.50;0.96)	0.73	5	0.71 (0.55;0.93)	0.79
Alzheimer	14	0.99 (0.91;1.07)	0.12	7	1.63 (1.06;2.49)	0.08	12	1.04 (0.97;1.10)	0.13	5	1.36 (0.98;1.90)	0.48	7	2.43 (1.93;3.06)	0.66	10	1.82 (1.54;2.16)	< 0.01
AMD	14	1.31 (1.22;1.39)	< 0.01	5	36.33 (18.91;69.81)	0.01	10	1.11 (1.03;1.20)	< 0.01	3	82.90 (31.41;218.81)	0.47	9	1.04 (0.84;1.29)	0.32	11	1.01 (0.86;1.18)	0.18

Note:

NA's occurred due to a lack of coverage, or when all variants were removed due to a high risk of horizontal pleiotropy; Effects presented as mean difference (OR) and 95% confidence intervals (95%CI) per unit change in protein or lipid concentration coded in the canonical direction.

Supplementary Table 7: Multivariable drug target Mendelian randomization. Assessing the potential lipid mediation of CETP or PCSK9 protein effects on clinical outcomes.

		CETP LDL-C	CETP HDL-C			PCSK9 LDL-C	PCSK9 HDL-C	
	No. variants	OR 95%CI	OR 95%CI	heterogeneity p-value	No. variants	OR 95%CI	OR 95%CI	heterogeneity p-value
CHD	15	0.73 (0.37;1.43)	0.85 (0.82;0.88)	0.12	8	0.66 (0.58;0.75)	1.42 (0.64;3.16)	0.37
Any stroke	15	0.97 (0.57;1.66)	1.03 (1.01;1.06)	0.04	8	0.80 (0.72;0.89)	0.62 (0.30;1.28)	0.69
Any ischemic stroke	15	0.87 (0.44;1.70)	1.05 (1.02;1.08)	0.18	8	0.81 (0.71;0.91)	0.68 (0.31;1.50)	0.80
Large artery stroke	14	1.46 (0.33;6.51)	1.15 (1.07;1.23)	< 0.01	8	0.68 (0.50;0.93)	0.19 (0.03;1.33)	0.60
Cardioembolic stroke	15	0.90 (0.28;2.89)	0.92 (0.87;0.97)	0.43	8	0.81 (0.59;1.10)	0.35 (0.05;2.45)	0.14
Small vessel stroke	13	1.29 (0.17;9.72)	0.93 (0.87;1.00)	< 0.01	8	0.89 (0.63;1.25)	0.18 (0.02;1.68)	0.18
HF	15	0.71 (0.45;1.12)	0.93 (0.91;0.95)	0.52	8	0.87 (0.79;0.95)	0.45 (0.24;0.83)	0.60
AF	15	0.68 (0.48;0.96)	0.94 (0.90;0.97)	0.49	8	0.86 (0.80;0.93)	1.20 (0.71;2.03)	0.63
T2DM	15	1.22 (0.81;1.84)	0.95 (0.93;0.97)	0.13	8	1.07 (0.98;1.17)	1.69 (0.90;3.16)	0.14
CKD	15	0.56 (0.33;0.96)	0.87 (0.84;0.89)	0.50	8	0.98 (0.88;1.10)	0.37 (0.17;0.80)	0.88
Asthma	15	0.32 (0.14;0.73)	0.90 (0.80;1.02)	0.68	8	1.43 (1.12;1.82)	5.81 (1.07;31.56)	0.88
IBD	15	1.91 (0.66;5.54)	1.12 (1.07;1.18)	0.33	8	0.95 (0.77;1.18)	0.44 (0.11;1.74)	0.62
CD	15	3.78 (0.98;14.64)	1.01 (0.95;1.08)	0.46	8	1.03 (0.75;1.42)	0.48 (0.07;3.53)	0.35
UC	15	1.25 (0.35;4.45)	1.20 (1.13;1.28)	0.52	8	1.08 (0.82;1.41)	0.48 (0.09;2.69)	0.47
MS	3	0.89 (0.01;69.94)	1.10 (0.90;1.34)	0.68	5	0.61 (0.43;0.87)	1.14 (0.14;9.16)	0.62
Alzheimer	13	0.76 (0.25;2.34)	0.94 (0.89;0.99)	0.27	7	2.39 (1.84;3.10)	2.89 (0.78;10.77)	< 0.01
AMD	11	3.40 (0.92;12.51)	1.72 (1.51;1.97)	< 0.01	8	1.00 (0.81;1.24)	1.50 (0.33;6.88)	0.25

Note:

Effects presented as odds ratio (OR) and 95% confidence intervals (95%CI) per SD change in lipid concentration (higher for HDL-C and lower for LDL-C).

Supplementary Table 8: Multivariable drug target Mendelian randomization. Assessing the potential Apo-B and HDL-C mediation of CETP protein effects on clinical outcomes.

		CETP Apo-B	CETP HDL-C	
	No. variants	OR 95%CI	OR 95%CI	heterogeneity p-value
CHD	16	0.60 (0.34;1.03)	0.93 (0.84;1.04)	0.02
Any stroke	15	0.63 (0.28;1.45)	1.13 (0.96;1.33)	0.07
Any ischemic stroke	15	0.64 (0.29;1.42)	1.14 (0.98;1.33)	0.24
Large artery stroke	14	0.12 (0.02;0.75)	1.74 (1.22;2.50)	< 0.01
Cardioembolic stroke	16	0.53 (0.15;1.86)	1.04 (0.81;1.33)	0.29
Small vessel stroke	14	0.28 (0.04;1.93)	1.21 (0.84;1.74)	0.01
HF	16	0.74 (0.46;1.19)	0.98 (0.89;1.07)	0.53
AF	16	0.79 (0.50;1.25)	0.97 (0.87;1.07)	0.27
T2DM	15	1.40 (0.88;2.24)	0.89 (0.82;0.98)	0.18
CKD	16	0.48 (0.24;0.93)	0.99 (0.86;1.13)	0.15
Asthma	15	0.27 (0.08;0.87)	1.12 (0.86;1.46)	0.46
IBD	16	0.95 (0.31;2.91)	1.15 (0.93;1.44)	0.23
CD	16	2.50 (0.61;10.27)	0.88 (0.66;1.16)	0.38
UC	16	0.41 (0.12;1.44)	1.44 (1.12;1.84)	0.48
MS	3	0.58 (0.03;10.35)	1.23 (0.65;2.35)	0.86
Alzheimer	14	1.52 (0.35;6.49)	0.86 (0.65;1.14)	0.16
AMD	12	2.11 (0.60;7.38)	1.52 (1.15;1.99)	< 0.01

Note:
Effects presented as odds ratio (OR) and 95% confidence intervals (95%CI) per SD change in lipid concentration (higher for HDL-C and lower for Apo-B).

Supplementary Table 9: CETP pQTL variants selected from within and around *CETP*; before pruning by minor allele frequency and clumping on linkage disequilibrium

rs-id	chromosome	position (build 37)	F-statistic
rs11076174	16	57003146	64.9
rs11076175	16	57006378	327.2
rs11076176	16	57007446	222.9
rs11508026	16	56999328	340.5
rs117040820	16	57005762	32.7
rs117427818	16	57010486	144.2
rs118146573	16	57000938	274.5
rs12444012	16	57001438	330.8
rs12447620	16	57014319	84.7
rs12447839	16	56993935	40.2
rs12447924	16	56994192	43.0
rs12597002	16	57002404	55.3
rs12708983	16	57014411	35.8
rs12708985	16	57014610	70.6
rs12720918	16	56994212	167.6
rs12720922	16	57000885	332.0
rs12720926	16	56998918	334.2
rs1532624	16	57005479	346.1
rs1532625	16	57005301	346.2
rs158477	16	57007610	36.7
rs158478	16	57007734	41.9
rs158479	16	57008048	34.3
rs158480	16	57008227	80.9
rs158617	16	57008287	96.5
rs17231506	16	56994528	442.9
rs1800775	16	56995236	354.7
rs1800777	16	57017319	99.6
rs1864163	16	56997233	341.9
rs1968905	16	57010948	34.6
rs289714	16	57007451	191.7
rs289717	16	57009388	101.7
rs289718	16	57009932	109.5
rs289719	16	57009941	109.5
rs289741	16	57017474	96.7
rs291043	16	57012699	101.0
rs291044	16	57011452	101.8
rs36229786	16	56993901	225.4
rs3816117	16	56996158	352.3
rs4369653	16	56997551	58.7
rs4783961	16	56994894	80.8
rs4783962	16	56995038	39.7
rs4784741	16	57001216	331.3
rs4784744	16	57011185	97.2
rs4784745	16	57014875	102.2
rs56208677	16	57010232	81.4
rs5882	16	57016092	87.5
rs60545348	16	57001985	55.3
rs708272	16	56996288	345.1
rs708273	16	56999949	56.1
rs711751	16	56993909	258.7

Supplementary Table 9: CETP pQTL variants selected from within and around *CETP*; before pruning by minor allele frequency and clumping on linkage disequilibrium *(continued)*

rs-id	chromosome	position (build 37)	F-statistic
rs711752	16	56996211	345.2
rs7203984	16	56999258	288.1
rs7205804	16	57004889	346.1
rs72771478	16	57002118	38.8
rs7499892	16	57006590	319.7
rs821840	16	56993886	411.0
rs9939224	16	57002732	321.1
rs36229491	16	56994245	449.8
rs34145065	16	56996646	349.5
rs5817082	16	56997350	298.8
rs17231569	16	56999779	334.2
rs12720908	16	57001255	330.7
rs200751500	16	57001275	325.1
rs201940645	16	57001580	219.8
rs200184680	16	57001581	306.9
rs756997703	16	57001582	330.5
rs35874588	16	57009652	111.3
rs12720941	16	57009658	101.3
rs200428946	16	57012560	73.0

Supplementary Table 10: LDL-C variants selected from within and around *CETP*; before pruning by minor allele frequency and clumping on linkage disequilibrium

rs-id	chromosome	position (build 37)	F-statistic
rs711751	16	56993909	22.5
rs17231506	16	56994528	123.1
rs4783961	16	56994894	47.3
rs1800775	16	56995236	101.1
rs35088638	16	56996158	76.2
rs711752	16	56996211	107.2
rs1864163	16	56997233	87.6
rs7203984	16	56999258	70.8
rs11508026	16	56999328	39.2
rs820299	16	57000284	16.0
rs12720922	16	57000885	54.7
rs118146573	16	57000938	41.8
rs12597002	16	57002404	18.7
rs9939224	16	57002732	29.9
rs11076174	16	57003146	21.7
rs7205804	16	57004889	111.9
rs1532625	16	57005301	82.0
rs1532624	16	57005479	113.3
rs11076175	16	57006378	60.8
rs7499892	16	57006590	76.5
rs289714	16	57007451	39.8
rs289719	16	57009941	24.7
rs289741	16	57017474	17.9

Supplementary Table 10: LDL-C variants selected from within and around *CETP*; before pruning by minor allele frequency and clumping on linkage disequilibrium *(continued)*

rs-id	chromosome	position (build 37)	F-statistic
rs59207181	16	57001584	78.4
rs186481539	16	56996647	72.1
rs34620476	16	56996649	77.1

Supplementary Table 11: HDL-C variants selected from within and around *CETP*; before pruning by minor allele frequency and clumping on linkage disequilibrium

rs-id	chromosome	position (build 37)	F-statistic
rs711751	16	56993909	619.2
rs12447839	16	56993935	150.1
rs12447924	16	56994192	238.1
rs17231506	16	56994528	1479.7
rs4783961	16	56994894	735.5
rs4783962	16	56995038	331.5
rs1800776	16	56995234	21.8
rs1800775	16	56995236	1479.7
rs35088638	16	56996158	1479.7
rs711752	16	56996211	1479.7
rs5030708	16	56996279	17.2
rs1864163	16	56997233	1479.7
rs7203984	16	56999258	1479.7
rs11508026	16	56999328	1454.8
rs820299	16	57000284	301.6
rs12720922	16	57000885	1455.7
rs118146573	16	57000938	1078.0
rs12597002	16	57002404	462.9
rs9939224	16	57002732	1193.4
rs11076174	16	57003146	576.4
rs7205804	16	57004889	1479.7
rs1532625	16	57005301	1479.7
rs1532624	16	57005479	1479.7
rs117040820	16	57005762	58.3
rs12720873	16	57006072	40.6
rs11076175	16	57006378	1479.7
rs7499892	16	57006590	1479.7
rs9930761	16	57007192	37.4
rs5883	16	57007353	136.3
rs289714	16	57007451	1479.7
rs289717	16	57009388	228.3
rs289719	16	57009941	786.2
rs2033254	16	57009985	19.0
rs4784744	16	57011185	220.9
rs891144	16	57011936	26.0
rs12708980	16	57012379	21.2
rs4784745	16	57014875	203.6
rs5882	16	57016092	259.5
rs9923854	16	57017002	63.6

Supplementary Table 11: HDL-C variants selected from within and around *CETP*; before pruning by minor allele frequency and clumping on linkage disequilibrium *(continued)*

rs-id	chromosome	position (build 37)	F-statistic
rs289741	16	57017474	725.7
rs1801706	16	57017662	64.3
rs59207181	16	57001584	1479.7
rs186481539	16	56996647	1462.8
rs192542140	16	56999353	20.8
rs34620476	16	56996649	1479.7

Supplementary Table 12: TG variants selected from within and around *CETP*; before pruning by minor allele frequency and clumping on linkage disequilibrium

rs-id	chromosome	position (build 37)	F-statistic
rs711751	16	56993909	22.8
rs17231506	16	56994528	79.2
rs4783961	16	56994894	32.4
rs1800775	16	56995236	114.0
rs35088638	16	56996158	61.2
rs711752	16	56996211	98.7
rs1864163	16	56997233	72.4
rs7203984	16	56999258	70.5
rs11508026	16	56999328	45.6
rs12720922	16	57000885	44.0
rs118146573	16	57000938	34.1
rs12597002	16	57002404	16.7
rs9939224	16	57002732	24.5
rs11076174	16	57003146	18.8
rs7205804	16	57004889	107.5
rs1532625	16	57005301	72.3
rs1532624	16	57005479	107.2
rs11076175	16	57006378	63.6
rs7499892	16	57006590	66.8
rs289714	16	57007451	41.0
rs59207181	16	57001584	58.7
rs186481539	16	56996647	54.7
rs34620476	16	56996649	57.5

Supplementary Table 13: PCSK9 pQTL variants selected from within and around *PCSK9*; before pruning by minor allele frequency and clumping on linkage disequilibrium

rs-id	chromosome	position (build 37)	F-statistic
rs11591147	1	55505647	72.2
NA	1	55520994	57.7
rs693668	1	55521109	54.7
rs472495	1	55521313	54.7
rs45448095	1	55505447	47.9

Supplementary Table 13: PCSK9 pQTL variants selected from within and around *PCSK9*; before pruning by minor allele frequency and clumping on linkage disequilibrium *(continued)*

rs-id	chromosome	position (build 37)	F-statistic
rs1165287	1	55520212	46.8
rs11583723	1	55505926	46.6
rs41294819	1	55504586	46.6
rs471705	1	55521242	46.1
rs11583680	1	55505668	45.7
rs373507733	1	55511372	44.7
rs479910	1	55522141	41.2
rs634272	1	55520265	41.1
rs521662	1	55519238	40.7
rs2495477	1	55518467	40.4
rs499883	1	55519174	40.0
rs71991605	1	55510813	38.8
rs4927193	1	55509872	37.9
rs45530931	1	55510971	37.9
rs7546522	1	55516713	37.4
rs639750	1	55519015	36.3
rs625619	1	55518166	33.9
rs2479409	1	55504650	33.4
rs494198	1	55518528	33.3
rs2479410	1	55505861	31.5
rs565436	1	55524601	29.5
rs11587071	1	55522674	29.0
rs535471	1	55514182	28.9
rs499718	1	55512549	28.9
rs534473	1	55514046	28.8
rs41294823	1	55514158	28.2
rs557211	1	55514215	28.2
rs479832	1	55515537	28.2
rs10585118	1	55515668	28.2
rs11310630	1	55522612	27.2
rs644000	1	55511995	27.1
rs557435	1	55520864	26.8
rs11436234	1	55511623	26.6
rs10888898	1	55516508	26.3
rs7530425	1	55511471	26.2
rs2483205	1	55518316	26.1
rs11206513	1	55507649	24.5

Supplementary Table 14: LDL-C variants selected from within and around *PCSK9*; before pruning by minor allele frequency and clumping on linkage disequilibrium

rs-id	chromosome	position (build 37)	F-statistic
rs17111503	1	55503448	200.8
rs2479409	1	55504650	222.5
rs11591147	1	55505647	647.3
rs11583680	1	55505668	32.4

Supplementary Table 14: LDL-C variants selected from within and around *PCSK9*; before pruning by minor allele frequency and clumping on linkage disequilibrium *(continued)*

rs-id	chromosome	position (build 37)	F-statistic
rs2479410	1	55505861	106.0
rs4927193	1	55509872	43.5
rs644000	1	55511995	108.8
rs499718	1	55512549	23.7
rs10888897	1	55513061	133.1
rs535471	1	55514182	17.6
rs11206514	1	55516004	142.0
rs572512	1	55517344	111.2
rs625619	1	55518166	56.0
rs2483205	1	55518316	84.1
rs7552841	1	55518752	61.1
rs557435	1	55520864	83.5
rs533375	1	55523361	145.7
rs584626	1	55523984	151.8
rs585131	1	55524116	153.7
rs540796	1	55524197	152.6
rs562556	1	55524237	88.1
rs568052	1	55524842	35.8
rs483462	1	55525400	49.5
rs602705	1	55525726	52.2
rs603247	1	55525868	55.5
rs615563	1	55526296	42.1
rs11206517	1	55526428	21.2
rs516499	1	55526685	61.0
rs630431	1	55527323	69.5
rs376826538	1	55527479	55.8
rs643257	1	55527918	57.0
rs12067569	1	55528629	72.2
rs505151	1	55529187	70.7
rs2495477	1	55518467	128.9

Supplementary Table 15: GWAS outcome data used in the drug target evaluations of CETP and PCSK9; ordered by number of events and total sample size.

Outcomes	No. Events	Sample size	URL
T2DM	74124	898130	https://www.nature.com/articles/s41588-018-0241-6
CHD	60801	184305	https://www.nature.com/articles/ng.3396
AF	60620	1030836	https://www.nature.com/articles/s41588-018-0171-3
HF	47309	977323	https://www.nature.com/articles/s41467-019-13690-5
CKD	41395	480698	https://www.ncbi.nlm.nih.gov/pmc/articles/PMC6698888/
Any stroke	40585	446696	http://www.megastroke.org/index.html
Any ischemic stroke	34217	440328	http://www.megastroke.org/index.html
IBD	32637	62341	https://www.ncbi.nlm.nih.gov/pubmed/23128233
Alzheimer	17008	74046	https://pubmed.ncbi.nlm.nih.gov/24162737/
AMD	16144	33976	https://www.nature.com/articles/ng.3448
MS	14498	38589	https://www.ncbi.nlm.nih.gov/pmc/articles/PMC3832895/
Cardioembolic stroke	7193	413304	http://www.megastroke.org/index.html
UC	6687	26405	https://www.nature.com/articles/ng.764
CD	5956	21770	https://www.nature.com/articles/ng.717
Small vessel stroke	5386	411497	http://europepmc.org/abstract/MED/20858683
Asthma	5135	30810	https://www.thelancet.com/journals/lanres/article/PIIS2213-2600(18)30389-8/fulltext
Large artery stroke	4373	410384	http://www.megastroke.org/index.html
Cognitive function	0	1131881	https://www.nature.com/articles/s41588-018-0147-3
DBP	0	1006863	https://pubmed.ncbi.nlm.nih.gov/30048462/
SBP	0	1006863	https://pubmed.ncbi.nlm.nih.gov/30048462/
BUN	0	480698	https://www.ncbi.nlm.nih.gov/pmc/articles/PMC6698888/
eGFR	0	480698	https://www.ncbi.nlm.nih.gov/pmc/articles/PMC6698888/
BMD	0	426824	https://www.nature.com/articles/s41588-018-0302-x
LDL-C	0	361194	http://www.nealelab.is/uk-biobank
HDL-C	0	361194	http://www.nealelab.is/uk-biobank
TG	0	361194	http://www.nealelab.is/uk-biobank
LP[a]	0	361194	http://www.nealelab.is/uk-biobank
ApoB	0	361194	http://www.nealelab.is/uk-biobank
ApoA1	0	361194	http://www.nealelab.is/uk-biobank
Glucose	0	361194	http://www.nealelab.is/uk-biobank
HbA1c	0	361194	http://www.nealelab.is/uk-biobank
Leukocytes	0	361194	http://www.nealelab.is/uk-biobank
Lymphocytes	0	361194	http://www.nealelab.is/uk-biobank
Monocytes	0	361194	http://www.nealelab.is/uk-biobank

Supplementary Table 15: GWAS outcome data used in the drug target evaluations of CETP and PCSK9; ordered by number of events and total sample size. *(continued)*

Outcomes	No. Events	Sample size	URL
Neutrophils	0	361194	http://www.nealelab.is/uk-biobank
Pulse rate	0	361194	http://www.nealelab.is/uk-biobank
CRP	0	361194	http://www.nealelab.is/uk-biobank

Note:
AF: Atrial Fibrillation, AMD: age-related macular degeneration, BMD: bone mineral density, BUN: blood urea nitrogin, CD: Crohn's disease, CHD: coronary heart disease, CKD: chronic kidney disease, CRP: c-reactive protein, DBP: diastolic blood pressure, eGFR: estimated glomerular filtration rate, HF: heart failure, IBD: Inflammatory bowel disease, MS: multiple sclerosis, SBP: systolic blood pressure, T2DM: type 2 diabetes, TG: triglycerides, UB: ulcerative colitis

Supplementary note 1

Medline

- exp Cholesteryl ester transfer protein inhibitor/
- Cholesteryl ester transfer protein inhibitor*.tw.
- Cholesterylester transfer protein inhibitor*.tw.
- CETP inhibitor*.tw.
- CETPI.tw.
- Anacetrapib.tw
- MK-0859.tw
- MK0859.tw
- ”MK 0859”.tw.
- Dalcetrapib.tw
- JTT-705.tw
- JTT705.tw.
- JTT 705.tw
- RO-4607381.tw
- Evacetrapib.tw
- LY2484595.tw
- LY 2484595.tw
- LY-2484595.tw.
- Obicetrapib.tw.
- TA-8995.tw
- AMG-899.tw.
- DEX-001.tw.
- Torcetrapib.tw
- CP-529414.tw
- CP-529,414.tw
- or/1-25
- exp Cholesteryl ester transfer protein/
- Cholesterylester transfer protein*.tw
- Cholesteryl ester transfer protein.tw
- Plasma lipid transfer protein*.tw
- CETP*.tw
- or/27-31
- exp Cardiovascular Diseases/
- cardio*.tw.
- cardia*.tw.
- heart*.tw.
- coronary*.tw.
- angina*.tw.
- ventric*.tw.
- myocard*.tw.
- pericard*.tw.
- isch?em*.tw.
- emboli*.tw.
- arrhythmi*.tw.
- thrombo*.tw.
- atrial fibrillat*.tw.
- tachycardi*.tw.
- endocardi*.tw.
- (sick adj sinus).tw.
- exp Stroke/
- (stroke or stokes).tw.
- cerebrovasc*.tw.
- cerebral vascular.tw.
- apoplexy.tw.
- (brain adj2 accident*).tw.
- ((brain* or cerebral or lacunar) adj2 infarct*).tw.
- exp Hyperlipidemias/
- hyperlipid*.tw.
- hyperlip?emia*.tw.
- hypercholesterol*.tw.
- hypercholester?emia*.tw.
- hyperlipoprotein?emia*.tw.
- hypertriglycerid?emia*.tw.
- exp Arteriosclerosis/
- exp Cholesterol/
- cholesterol.tw.
- “coronary risk factor* ”.tw.
- or/31-67
- 26 and 32 and 68
- randomi?ed controlled trial.pt.
- controlled clinical trial.pt.
- randomi?ed.ab.
- placebo.ab.
- drug therapy.fs.
- randomly.ab.
- trial.ab.
- groups.ab.
- 70 or 71 or 72 or 73 or 74 or 75 or 76

or 77
- exp animals/ not humans.sh.
- 78 not 79
- 69 and 80

EMBASE

- 'Cholesteryl ester transfer protein inhibitor'/exp
- Cholesteryl ester transfer protein inhibitor*
- Cholesterylester transfer protein inhibitor*
- CETP inhibitor*
- CETPI
- Anacetrapib
- MK-0859
- MK0859
- "MK 0859"
- Dalcetrapib
- JTT-705
- JTT705
- JTT 705
- RO-4607381
- Evacetrapib
- LY2484595
- LY 2484595
- LY-2484595
- Obicetrapib
- TA-8995
- AMG-899
- DEX-001
- Torcetrapib
- CP-529414
- CP-529,414
- 1 OR 2 OR 3 OR 4 OR 5 OR 6 OR 7 OR 8 OR 9 OR 10 OR 11 OR 12 OR 13 OR 14 OR 15 OR 16 OR 17 OR 18 OR 19 OR 20 OR 21 OR 22 OR 23 OR 24 OR 25
- 'Cholesteryl ester transfer protein'/exp
- Cholesterylester transfer protein*
- Cholesteryl ester transfer protein
- Plasma lipid transfer protein*
- CETP*
- 27 OR 28 OR 29 OR 30 OR 31
- 'cardiovascular disease'/exp
- cardio*
- cardia*
- heart*
- coronary*
- angina*
- ventric*
- myocard*
- pericard*
- isch?em*
- emboli*
- arrhythmi*
- thrombo*
- atrial fibrillat*
- tachycardi*
- endocardi*
- sick NEXT/1 sinus
- 'cerebrovascular disease'/exp
- stroke or stokes
- cerebrovasc*
- cerebral vascular
- apoplexy
- brain NEXT/3 accident*
- (brain* or cerebral or lacunar) NEXT/3 infarct*
- exp hyperlipidemia
- hyperlipid*
- hyperlip?emia*
- hypercholesterol*
- hypercholester?emia*
- hyperlipoprotein?emia*
- hypertriglycerid?emia*
- 'Arteriosclerosis'/exp
- 'Cholesterol'/exp
- cholesterol
- "coronary risk factor*"
- 33 OR 34 OR 35 OR 36 OR 37 OR 38 OR 39 OR 40 OR 41 OR 42 OR 43 OR 44 OR 45 OR 46 OR 47 OR 48 OR 49 OR 50 OR 51 OR 52 OR 53 OR 54 OR 55 OR 56 OR 57 OR 58 OR 59 OR 60 OR 61 OR 62 OR 63 OR 64 OR 65 OR 66 OR 67

- 26 and 32 and 68
- random$
- factorial$
- crossover$
- cross over$
- cross-over$
- placebo$
- doubl$ NEXT/1 blind$
- singl$ NEXT/1 blind$
- assign$
- allocat$
- volunteer$
- crossover procedure
- double blind procedure
- randomi?ed controlled trial
- single blind procedure
- 70 OR 71 OR 72 OR 73 OR 74 OR 75 OR 76 OR 78 OR 79 OR 80 OR 81 OR 82 OR 83 OR 84
- (animal or nonhuman) not human
- 85 not 86
- 69 and 87
- 88 AND [embase]/lim

Supplementary note 2

CETP inhibitor dose-response and compound potency

In-silico IC50 measurments, representing the dosage neccesary for 50% inhibition, were extracted from Chembl (v25). The IC50 measurement were relatively comparable between anacetrapib (7-60 nmol/L ;n=5), evacetrapib (26-110 nmol/L; n=2) and torcetrapib (3-160 nmol/L; n=8). For dalcetrapib these measured were considerably higher: 4100-6000 nmol/L (N=2).

The four included CETP inhibitors all showed a dose response relation with high-density lipoprotein cholesterol (HDL-C). Evacetrapib had the strongest response, with HDL-C increasing between 54% and 129% (from baseline) at escalating doses of 30-500 mg per day[1]. Torcetrapib increased HDL-C by 16% to 91% (10-240 mg per day)[2], followed by anacetrapib: 9% to 48% (10-300 mg per day)[3]. At 4% to 34% for 300-900 mg per day[4], dalcetrapib, while still comparable to anacetrapib, showed the weakest dose response.

In general the difference in potency and dose-response were minimal between anacetrapib, evacetrapib, and torcetrapib, and were prescribed to a similar dosage in phase 3 RCT. The clear difference of dalcetrapib, which was considerably less potent, resulting in a marked increase in daily dosage, between 600 and 900 mg.

Supplementary Table 16: The preclinical drug potency (IC50), drug response in phase 1 clinical studies and the final choice of drug dose in phase 3 RCT

Drug	Potency (IC50)		Phase 1		Phase 3
	Mean (nmol/L)	Range (nmol/L)	Dose (mg)	%Δ HDL-C (mg)	Dose
Anacetrapib	36	17 to 60	10 to 300	9 to 48	100
Dalcetrapib	5050	4100 to 6000	300 to 900	4 to 34	600 to 900
Evacetrapib	68	26 to 110	30 to 500	54 to 129	130
Torcetrapib	52	3 to 160	10 to 240	16 to 91	60 to 120

Supplementary references

[1] Stephen J. Nicholls et al. “Effects of the CETP Inhibitor Evacetrapib Administered as Monotherapy or in Combination With Statins on HDL and LDL Cholesterol: A Randomized Controlled Trial.” In: *JAMA* 306.19 (2011), pp. 2099–2109. DOI: `10.1001/jama.2011.1649`.

[2] Ronald W. Clark et al. “Raising high-density lipoprotein in humans through inhibition of cholesteryl ester transfer protein: an initial multidose study of torcetrapib.” In: *Arterioscler. Thromb. Vasc. Biol.* (2004).

[3] Hayes M. Dansky et al. “Efficacy and safety after cessation of treatment with the cholesteryl ester transfer protein inhibitor anacetrapib (MK-0859) in patients with primary hypercholesterolemia or mixed hyperlipidemia.” In: *American Heart Journal* 162.4 (2011), pp. 708 –716. DOI: `https://doi.org/10.1016/j.ahj.2011.07.010`.

[4] Greetje J. De Grooth et al. “Efficacy and safety of a novel cholesteryl ester transfer protein inhibitor, JTT-705, in humans: a randomized phase II dose-response study.” In: *Circulaton* (2002).
