# Supplementary material for: Cholesteryl ester transfer protein (CETP) as a drug target for cardiovascular disease
Source: Nat Commun. 2021 Sep 24;12:5640. doi: 10.1038/s41467-021-25703-3 (PMC8463530; doi:10.1038/s41467-021-25703-3)
Supplement: Supplementary file 3 — Reporting Summary [file 41467_2021_25703_MOESM3_ESM.pdf]

nature research

Corresponding author(s): Schmidt AF

Last updated by author(s): Aug 5, 2021

Reporting Summary

Nature Research wishes to improve the reproducibility of the work that we publish. This form provides structure for consistency and transparency in reporting. For further information on Nature Research policies, see our Editorial Policies and the Editorial Policy Checklist.

Statistics

For all statistical analyses, confirm that the following items are present in the figure legend, table legend, main text, or Methods section.

n/a	Confirmed	
☐	☒	The exact sample size (n) for each experimental group/condition, given as a discrete number and unit of measurement
☐	☒	A statement on whether measurements were taken from distinct samples or whether the same sample was measured repeatedly
☐	☒	The statistical test(s) used AND whether they are one- or two-sided<br>*Only common tests should be described solely by name; describe more complex techniques in the Methods section.*
☒	☐	A description of all covariates tested
☐	☒	A description of any assumptions or corrections, such as tests of normality and adjustment for multiple comparisons
☐	☒	A full description of the statistical parameters including central tendency (e.g. means) or other basic estimates (e.g. regression coefficient) AND variation (e.g. standard deviation) or associated estimates of uncertainty (e.g. confidence intervals)
☐	☒	For null hypothesis testing, the test statistic (e.g. F, t, r) with confidence intervals, effect sizes, degrees of freedom and P value noted<br>*Give P values as exact values whenever suitable.*
☒	☐	For Bayesian analysis, information on the choice of priors and Markov chain Monte Carlo settings
☒	☐	For hierarchical and complex designs, identification of the appropriate level for tests and full reporting of outcomes
☐	☒	Estimates of effect sizes (e.g. Cohen's d, Pearson's r), indicating how they were calculated

Our web collection on statistics for biologists contains articles on many of the points above.

Software and code

Policy information about availability of computer code

Data collection	Data were collected from publicly available GWAS repositories; no specialist software was used for this.
Data analysis	Analyses were conducted using Python v3.7.4 (for GNU Linux), Pandas v0.25, Numpy v1.15, Seaborn v0.11.5, R v4.0. 3 (for GNU Linux), ggplot2 v3.3.5, metafor58 v3.0.2 and forestplot v1.10.1. The Python and R scripts, and data necessary to generate the illustrations have been deposited through the UCL Research Data Repository : https://doi.org/10.5522/04/13686247.v3.

For manuscripts utilizing custom algorithms or software that are central to the research but not yet described in published literature, software must be made available to editors and reviewers. We strongly encourage code deposition in a community repository (e.g. GitHub). See the Nature Research guidelines for submitting code & software for further information.

Data

Policy information about availability of data

All manuscripts must include a data availability statement. This statement should provide the following information, where applicable:
- Accession codes, unique identifiers, or web links for publicly available datasets
- A list of figures that have associated raw data
- A description of any restrictions on data availability

Source data are provided with this paper, specifically the MR and trial results, as well as the data underlying each figure have been deposited through the UCL Research Data Repository: https://doi.org/10.5522/04/13686247.v3 . The supplemental tables include all the aggregated data (effect estimates, standard errors, and so on) presented here. All source GWAS data are publicly available (see URL supplied in Supplementary Table 15) including 60,801 CHD cases from CardiogramplusC4D38 (http://www.cardiogramplusc4d.org/); 40,585 stroke cases (subtypes) from MEGASTROKE39 (http://www.megastroke.org/index.html); 47,309 HF cases from HERMES40 (https://www.ebi.ac.uk/gwas/publications/31919418), 60,620 atrial fibrillation cases from AFgen41 (http://csg.sph.umich.edu/willer/public/afib2018/), 17,008 Alzheimer's disease42 cases(https://www.niagads.org/), 16,144 age-related macular degeneration events from IAMDGC43,44

(http://amdgenetics.org/), and genetic associations with NMR measured circulating lipoprotein subfractions and other metabolites were available from a meta-analysis of Kettunen et al.45, and UCLEB46 (n: 33,029, http://www.computationalmedicine.fi/data/NMR_GWAS/). Additionally, the following resources were sourced: major circulating lipid sub-fractions or apolioproteins (LDL-C, HDL-C, triglycerides, lipoprotein A [Lp(a)], apolipoprotein B, apolipoprotein A1), pulse rate, glucose and HbA1c, leukocytes, lymphocytes, monocytes, neutrophil counts, and C-reactive protein, using data from the UK biobank (UKB - http://www.nealelab.is/uk-biobank). Blood pressure (systolic and diastolic) data were available from Evangelou et al.47 (https://grasp.nhlbi.nih.gov/FullResults.aspx).Carotid artery intima media thickness was available from a meta-analysis of the Cohorts for Aging Research in Genomic Epidemiology (CHARGE)48 and University College London Edinburgh Bristol (UCLEB)46 (https://www.ncbi.nlm.nih.gov/projects/gap/cgi-bin/study.cgi?study_id=phs000930.v6.p1). The CKDGen consortium provided GWAS associations on blood urea nitrogen (BUN), estimated glomerular filtration rate (eGFR), and chronic kidney disease49 (http://ckdgen.imbi.uni-freiburg.de/). Bone mineral density50 GWAS data were obtained from GEFOS Consortium (http://www.gefos.org/). Genetic associations with “general cognitive function” were obtained from a meta-analysis of CHARGE, COGENT and UKB51 (https://www.thessgac.org/data). Data were extracted on type 2 diabetes52 from DIAGRAM (http://diagram-consortium.org/index.html); asthma53 (ftp://ftp.ebi.ac.uk/pub/databases/gwas/summary_statistics/GCST006001-GCST007000/GCST006911), inflammatory bowel disease54, Crohn’s disease55 and ulcerative colitis56 from IIBDGC (https://www.ibdgenetics.org/); multiple sclerosis57 from the IMSG consortium (https://imsgc.net/). Finally, genetic association with CETP or PCSK9 concentration were sourced from Blauw et al14 (https://www.ahajournals.org/doi/full/10.1161/CIRCGEN.117.002034) and Plot et al37 (https://pubmed.ncbi.nlm.nih.gov/29748315/).

Field-specific reporting

Please select the one below that is the best fit for your research. If you are not sure, read the appropriate sections before making your selection.

☒ Life sciences ☐ Behavioural & social sciences ☐ Ecological, evolutionary & environmental sciences

For a reference copy of the document with all sections, see nature.com/documents/nr-reporting-summary-flat.pdf

Life sciences study design

All studies must disclose on these points even when the disclosure is negative.

Sample size	The manuscript represent meta-analyses of publicly available trial data in the form of point estimates and standard errors, and drug target MR analyses using similarly publicly available data on the genetic association of CETP and PCSK9 variants with protein concentration and clinically relevant traits. This re-use of publicly available data provides an important opportunity to gain additional insights without additional burden or risk to participants. As such we did not perform any de novo participant recruitment, nor related sample size calculations. Precision of our results is indicated by 95% confidence intervals, where wide confidence intervals provide a clear indication results can be improved by increasing sample size beyond the currently available number.
Data exclusions	To improve coomputational stability, low MAF (below 0.01) variants were excluded (a priori defined).
Replication	The presented drug target Mendelian randomization analyses sourced publicly available data on the genetic association with CETP concentration or with PCSK9 concentration from:<br>Blauw et al: https://pubmed.ncbi.nlm.nih.gov/29728394/<br>Pott et al: https://pubmed.ncbi.nlm.nih.gov/29748315/<br><br>The CETP drug target analysis was replicated using three proxies of protein concentration and activity. Specifically, we used genetic variants associated with LDL-C, HDL-C, or TG selected form the same 2.5 kb flanking region around CETP used to identify genetic instruments for protein concentration from Blauw et al. The PCSK9 analysis was replicated using genetic association with LDL-C selected from the same 2.5 kb PCSK9 flanking region used to identify genetic association with protein concentration from Pott et all.
Randomization	The manuscripts includes a meta-analysis of randomized controlled trials, were subjects were randomly allocated to CETP inhibition or placebo groups. Furthermore, we conducted drug target mendelian randomization, where – following Mendel law’s of inheritance, genetic variants are assumed to be randomly allocated during gamete formation, resulting in a naturally occurring randomized experiment.
Blinding	The manuscript describes a meta-analyses of aggregated data (point estimates and standard errors) of blinded placebo controlled trials, where both participants and study personal were blinded for treatment allocation. The current researchers were not involved with these trials and hence had no knowledge of allocation.<br>Additionally, we performed drug target Mendelian randomization leveraging aggregated genetic associations (again point estimates and standard errors) with CETP or PCSK9 concentration to anticipate effects of inhibiting these drug targets would have clinically relevant outcomes. Following Mendel’s laws of inheritance genetic loci are inherited randomly, resulting in a natural experiment or pseudo randomized experiment. In this setting the exposure of interest is the genetic variant encoding a drug target; both the research and the subjects were unaware of their genotype.

Reporting for specific materials, systems and methods

We require information from authors about some types of materials, experimental systems and methods used in many studies. Here, indicate whether each material, system or method listed is relevant to your study. If you are not sure if a list item applies to your research, read the appropriate section before selecting a response.

Materials & experimental systems

n/a	Involved in the study
☒	☐ Antibodies
☒	☐ Eukaryotic cell lines
☒	☐ Palaeontology and archaeology
☒	☐ Animals and other organisms
☒	☐ Human research participants
☒	☐ Clinical data
☒	☐ Dual use research of concern

Methods

n/a	Involved in the study
☒	☐ ChIP-seq
☒	☐ Flow cytometry
☒	☐ MRI-based neuroimaging
